# Hepatocellular Carcinoma in 2021: An Exhaustive Update

**DOI:** 10.7759/cureus.19274

**Published:** 2021-11-05

**Authors:** Cyriac A Philips, Sasidharan Rajesh, Dinu C Nair, Rizwan Ahamed, Jinsha K Abduljaleel, Philip Augustine

**Affiliations:** 1 Clinical and Translational Hepatology, The Liver Institute, Center of Excellence in GI Sciences, Rajagiri Hospital, Aluva, IND; 2 Interventional Hepatobiliary Radiology, Center of Excellence in GI Sciences, Rajagiri Hospital, Aluva, IND; 3 Gastroenterology and Advanced Gastrointestinal (GI) Endoscopy, Center of Excellence in GI Sciences, Rajagiri Hospital, Aluva, IND

**Keywords:** tace, bclc, ablation, liver transplantation, chronic hepatitis, cirrhosis, portal hypertension, liver cancer

## Abstract

Primary liver cancer is a challenging global health concern with an estimated more than a million persons to be affected annually by the year 2025. The commonest type is hepatocellular carcinoma (HCC), which has been increasing in incidence the world over, mostly due to chronic viral hepatitis B infection. In the last decade, paradigm changes in the etiology, understanding of molecular biology, and pathogenesis, including the role of gut microbiota; medical and surgical treatments, and outcome trends are notable. The application of omics-based technology has helped us unlock the molecular and immune landscape of HCC, through which novel targets for drug treatment such as immune-checkpoint inhibitors have been identified. Novel tools for the surveillance and diagnosis of HCC include protein-, genomics-, and composite algorithm-based clinical/biomarker panels. Magnetic resonance imaging-based novel techniques have improved HCC diagnosis through ancillary features that enhance classical criteria while positron emission tomography has shown value in prognostication. Identification of the role of gut microbiota in the causation and progression of HCC has opened areas for novel therapeutic research. A select group of patients still benefit from modified surgical and early interventional radiology treatments. Improvements in radiotherapy protocols, identification of parameters of futility among radiological interventions, and the emergence of novel first-line systemic therapies that include a combination of antiangiogenic and immune-checkpoint inhibitors have seen a paradigm change in progression-free and overall survival. The current review is aimed at providing exhaustive updates on the etiology, molecular biology, biomarker diagnosis, imaging, and recommended treatment options in patients with HCC.

## Introduction and background

Liver malignancy is a challenging global health concern with increasing incidence worldwide. Estimations reveal that by the year 2025, more than a million persons will be affected with liver cancer annually. Chronic hepatitis B virus (HBV) infection is the leading cause in approximately 50% of the cases of HCC, whereas currently, non-alcohol-related steatohepatitis (NASH) is rapidly becoming a growing etiological concern. The annual rate of HCC in Child-Turcotte-Pugh class A or B alcohol-associated cirrhosis is approximately 2.5%. A consensus on the rate of development of HCC in Child-Pugh C cirrhosis patients remains unavailable due to low cumulative survival in this group, differences in liver transplantation decisions (early vs late) globally, and lack of adequate data in the pre-transplant period. Apart from the changing etiology of HCC, the role of specific genotoxins such as aristolochic acid (in flowering birthwort plants, Aristolochia, and wild ginger; commonly used in traditional Chinese medicine), aflatoxin B1 (urinary excretion of aflatoxin metabolites impose 4-fold increased risk), and tobacco have been identified as pathogenetic cofactors and that of coffee and aspirin as preventive factors for the development of liver cancer [1-2]. The pathophysiology and molecular pathogenesis of HCC according to the genotoxicity and associated etiologies have come a long way, waiting to be fully translated to clinical practice. Classically, HCC is diagnosed via noninvasive criteria and the treatment chosen, depending on the overall tumor burden and underlying liver disease severity. However, novel evidence points towards the importance of histology and molecular characterizations that drive pathogenesis that provides novel druggable targets [1,3]. Similarly, with respect to the treatments offered, even though liver transplantation (LT) remains curative in well-selected patients and local ablation with radiofrequency (RFA) remain first-line treatments for small HCC, in those with intermediate and advanced HCC, the upcoming role of various immunomodulatory systemic therapies, transarterial radioembolization (TARE), and the use of various ‘safer’ radiation oncology techniques that increase transplant-free survival or downstaging to LT are demonstrably hopeful [3-4]. In this exhaustive paper, we review current updates on the etiology and epidemiology of HCC, discuss newly identified pathogenetic mechanisms, novel biomarkers, and prognostic tools, and apprise current and new treatment options for this calamitous disease.

## Review

Updates on epidemiology, etiology, and surveillance

Cancer of the liver is the sixth most diagnosed malignancy and the fourth leading cause of cancer-related death worldwide, with the highest incidence and mortality in East Asia (China, Japan, Mongolia, North Korea, South Korea, and Taiwan) and Africa. HCC is also the fifth most common cancer in men and the seventh in women globally. The global incidence of HCC is diverse due to the variable prevalence of associated etiologies, for example, 72% of cases occur in Asia with >50% of those in China alone, while in North America, it is 5%. In East Asia, the highest age-standardized incidence (ASIR per 100,000 persons) as well as mortality (ASMR) rate of HCC is notable in Mongolia. Strikingly, globally, ASIR and ASMR are approximately equal, showcasing the high fatalities associated with HCC [1-3,5]. Cirrhosis is the most common primary risk factor for HCC with one-third of patients developing liver cancer during their lifetime with an annual incidence of up to 8% on long-term follow-up. Even though the incidence of HCC in alcohol-associated and nonalcoholic fatty liver disease is lower when compared to chronic viral hepatitis, alcohol-associated cirrhosis is the second-most common cause for incident cases of HCC worldwide, following HBV virus infection [5]. An increased risk of developing HCC has been reported in most parts of the world, except in Northern Europe, due to the presence of multiple etiologies of liver cirrhosis development (for example, chronic viral hepatitis and alcohol use). A recent publication projecting HCC occurrence in 30 countries worldwide, also predicts the percentage change in ASIR over a 25-year period, from 2005 to 2030. The largest increases among men were predicted in Norway and among female North American blacks. On the other hand, a decrease in liver cancer among men and women was predicted to occur in Japan. The study also predicted a drop in the incidence of HCC attributable to chronic viral hepatitis, with an increased incidence of NASH-related HCC by 122% between 2016 and 2030 in the United States, demonstrating the importance of imposing preventive measures to curb damaging outcomes in this group of liver disease patients [6-7]. China accounted for a third of the worldwide cancer cases attributable to infection, due to the high ASIR of H pylori and hepatitis B virus infection [8]. In patients with HBV, in the absence of chronic liver disease, surveillance for HCC is warranted, especially in those with high viral load, envelope antigen positivity, family history of HCC, and genotype C. However, in patients with chronic hepatitis C virus infection, surveillance was found to be cost-effective only when cirrhosis was confirmed (liver stiffness measurement; fibrosis grade > 3). A recent large study demonstrated that the risk of HCC was significantly higher among those with acute hepatitis D virus (HDV) infection (relative risk, RR 6.1) or chronic HDV infection (RR 3.9) than in those with only HBV infection [9].

From an etiology point of view, it is important to realize that nonalcoholic fatty liver disease (NAFLD) and nonalcoholic steatohepatitis (NASH) are rising in rank as contributors to HCC development. Approximately 10-30% of NAFLD progresses to cirrhosis, with an annual incidence of HCC likely to be 1-2%, and several cohort studies showed that over 25% of NASH-related HCC can occur in the absence of cirrhosis - nonetheless, the actual impact of metabolic syndrome on the epidemiology of HCC remains underestimated. A study showed that a one-unit increase in body mass index (BMI) z-score at age seven or 13 years was associated with a 20-30% increased risk of liver cancer and obesity in early adulthood and was associated with a two to three-fold increased risk for HCC [10-11]. In 2018, the estimated global incidence rate of liver cancer per 100,000 person-years was 9.3 while the corresponding mortality rate was 8.5, incidence rates among men are two to four-fold higher than women, and the greatest gender differences are seen in Europe (e.g., France and Malta) where rates among men are greater than four-fold higher than in women [12-14].

Factors that have been consistently shown to promote the prevention of HCC among the general population and chronic liver disease patients include coffee consumption, aspirin use, and metformin use, with the highest evidence for coffee use (the risk of HCC reduced by 40% for any coffee consumption vs no consumption). Evidence from two population-based studies demonstrated that statin use was associated with a 39% lower risk of liver cancer, which was notable in HCC but not in intrahepatic bile duct carcinoma [15-18]. High-quality metanalyses and systematic reviews have shown that HCC risk, even though not fully eliminated, is reduced up to 80% in patients with hepatitis C virus (HCV)-related cirrhosis who attain sustained viral response (SVR) after the use of direct-acting antivirals. Nonetheless, among those who achieved SVR, HCC risk was still high in the presence of continued alcohol use, older age, infection with HCV genotype 3, and elevated hepatic fibrosis markers [19-20].

The current surveillance protocol for detecting HCC in cirrhosis of ultrasound (overall sensitivity for HCC detection, 84%; sensitivity for detection of early HCC, 47%) liver every six months was derived from a randomized controlled Chinese study on 18,000 patients with HBV-related liver disease, which may not hold true for other etiologies or in patients with multiple etiologies for cirrhosis. Nonetheless, further studies, mostly retrospective and some prospective observational, inclusive of lead-time and length-time biases, have shown that six-monthly screenings for HCC detection still held true for real-time clinical practice. Nevertheless, a recent prospective cohort study comparing magnetic resonance imaging (MRI)-based and ultrasound-based surveillance in predominantly HBV cirrhosis found that the former modality had a significantly higher sensitivity for early HCC detection. However, further data about MRI performance in non-HBV patients, cost-effectiveness, potential physical harms, and limited radiologic capacity need to be addressed in future studies to agree on routine use [21-22].

In recent times, the use of alternate imaging strategies as part of surveillance such as the abbreviated MRI protocol (the option including non-contrast, dynamic, or hepatobiliary phase post-gadoxetate injection) has shown high sensitivity for early HCC detection approaching that of diagnostic MRI. A meta-analysis showed that the sensitivity of abbreviated MRI for detection of HCC < 2 cm was lower than that for HCC ≥ 2 cm while the sensitivity and specificity of non-contrast abbreviated MRI were comparable to contrast-enhanced abbreviated MRI [23].

The use of a combination of biomarkers and radiological methods was found superior to either alone for HCC surveillance. In this regard, alpha-fetoprotein (AFP) and concomitant ultrasound improved early detection of HCC with a sensitivity of 63% and specificity of 84%. Trend in AFP levels and not the absolute single time value was found to have better predictability in diagnosing early HCC from a surveillance point of view, i.e., patients with consistent increments in AFP, even when below 20 ng/mL need close, attentive surveillance. The standard cut-off of 20 ng/mL for AFP for HCC detection was derived from patients with viral hepatitis; further, the use of different AFP cutoffs according to the liver disease etiology may improve specificity - higher cut-off of 59 ng/mL in patients with cirrhosis from viral hepatitis and lower cut-off of 11 ng/mL in those with non-viral etiology of cirrhosis [24]. Other recently published surveillance tools (discussed in more detail in subsequent sections) include the HCC early detection screening model, which utilized serial AFP measures to showcase the rate of change in AFP along with age, alanine aminotransferase (ALT), and platelet counts; GALAD model, which includes gender, age, AFP-L3 % (third electrophoretic form of lentil lectin-reactive AFP), AFP, and des-Gamma-Carboxy Prothrombin (DCP); the AFP, fucosylated kininogen, age, gender, alkaline phosphatase, and ALT model and methylated DNA marker panel, all which require further validation from independent groups and in specific cirrhosis etiologies [25-26].

Future improvisation to improve on the early detection of HCC in cirrhosis has led to the development of large prospective cohorts with stored biobanks of longitudinal serum and plasma samples such as the Early Detection Research Network (EDRN), Hepatocellular cancer Early Detection Study (HEDS), and Cancer Prevention Research Institute of Texas (CPRIT) Texas HCC Consortium (THCCC) cohorts, and the European network, STHEPBIO consortium, which would move into phase III validation of candidate biomarkers [27-30].

Updates on molecular biology, mechanisms, and classifications

The pathogenesis, etiology, and classification of HCC evolved from its original HBV-associated HCC in 1981 to the role of HCV in 1989, the contribution of p53 mutation in 1996, the use of noninvasive criteria for definition and classification in early 2000, to the association of NASH and HCC in 2002-2003. Various staging systems, based on these classifications, evolved from the original Okuda staging in 1985, which was replaced in 1999 by the Barcelona Clinic Liver Cancer (BCLC) staging system, mostly used in the Western world, and the Hong-Kong staging system (2014), used in more recent years in Asia. Current evidence sheds light on novel aspects in the complex multistep process in the development and progression of HCC, which include the interplay of various factors such as genetic predisposition, interactions between host, viral, and nonviral factors, cellular microenvironmental modulation, the role of immune cells, and, finally, the severity and etiology of the underlying liver disease [31].

The cell of origin for the development of HCC has been classically considered the adult hepatocyte, which directly transforms into HCC cells through a series of genetic alterations; or dedifferentiates into hepatocyte precursors that have progenitor cell markers; or transdifferentiates into biliary-like cells. The latter two processes can give rise to a combined tumor, such as hepato-cholangiocarcinoma, or produce intrahepatic cholangiocarcinoma [32-33].

Through next-generation sequencing, genomic profiling technology, and advanced computational methods, molecular pathogenetic mechanisms for distinguishing between driver and passenger mutations have allowed researchers to identify the main genes causally implicated in HCC development. These genes are categorized into major biological pathways such as telomere maintenance, Wnt/β-catenin, P53/cell cycle regulation, AKT/mTOR, MAP kinase, epigenetic modifiers, and those associated with oxidative stress. In HCC, the most prevalently mutated genes are telomerase reverse transcriptase (TERT), promoter, tumor protein (TP)53, catenin-β gene-1 (CTNNB1), AXIN1, ARID1A, and ARID2. Based on the interplay between mutated driver genes (epistatic interactions), three main clusters of positive interactions were described. The first between CTNNB1 mutation and TERT promoter, ARID2 and NFE2L2 mutations; the second between AXIN1 mutation and RPS6KA3 and ARID1A mutations; the third between TP53 mutation and KEAP1, and TSC2 mutation and CCND1/FGF19 amplification. Furthermore, a negative interaction was identified between CTNNB1 mutations and AXIN1 and TP53 mutations [34-35].

Established on these findings, HCC is now broadly classified into proliferation and nonproliferation (inflammation) types. The proliferation class is further subdivided into two subclasses and respective histological subtypes - the ‘Wnt-TGFβ’ subclass is characterized by activation of both TGFβ and Wnt pathways and an exhausted immune response, and a ‘progenitors’ subclass defined by the transcriptional and protein overexpression of hepatic progenitor markers. Tumors belonging to the proliferation group are highly heterogeneous, enriched in signaling pathways (e.g., transforming growth factor-β, phosphatidylinositol 3-kinase, and protein kinase B (PI3K-Akt), insulin-like growth factor, mechanistic target of rapamycin (mTOR), with stem-cell signaling Notch) and with very high gene expression patterns that result in tumor recurrence and shorter patient survival. These tumors also harbor aberrant epigenetic expression patterns of microRNAs and DNA methylation, which are also linked to chromosomal aberrations (commonly in chromosomes one and eight). On the other hand, the non-proliferating HCC most often has retained hepatocyte-like features, subset activation of the canonical Wnt, epidermal growth factor signaling, and activation of inflammation pathways, such as nuclear factor-kB and interleukin (IL)-6; mutations in the CTNNB1 gene tend to be less-aggressive, well-differentiated tumors with low AFP secretion and good prognosis. Nevertheless, the most frequent mutations in HCC are un-targetable, including those in the TERT promoter (60%-70%), TP53 (25%-50%), and CTNNB1. Overall, approximately one one-fourth of patients with HCC have at least one potential actionable mutation as per current standards [36]. The updated molecular and immunological classification of HCC is shown in Table 1.

**Table 1:** Components and pertinent features of molecular and immune classification of hepatocellular carcinoma AFP: alpha-fetoprotein; FLC: fibrolamellar carcinoma; HCC: hepatocellular carcinoma

Parameters	Type of hepatocellular carcinoma	
	Proliferation class	Non-proliferation / Inflammatory class
Molecular subclass	Cluster A/high proliferation S1/iCluster 3 – TGFβ Wnt – G2, G3 class S2/ iCluster 2 – progenitor type	Cluster B / S3 / iCluster 2 G4 class – interferon, Poly7 subtypes WNT/β catenin, CTNNB1 type – G5, G6 class
Pathological subclass	Progenitor type – mixed FLC/HCC, Macrotrabecular (G3) – massive	Steatohepatic HCC (G4 subtype), Cholestatic HCC (G5, G6)
Immunohistochemistry	Both - Phosphor-RPS6, Mixed FLC/HCC - Stem cell marker, CK19+, and EPCAM, pERK+	Steatotic type – CRP+ Cholestatic type – GS+, nuclear β catenin
Cell differentiation	Poor	Well to moderately differentiated (more hepatocyte-like)
Etiological association	HBV	Alcohol, HCV, NASH
Chromosomal characteristics	Chromosomal instability FGF19 / CCND1 amplification 17p loss	Chromosomal stability, Chromosome 7 amplification
Genetic features	TERT promoter mutation, TP53 mutation, Progenitor type – AXIN1, RPS6Ka3 mutation, Macrotrabecular type – TSC1-TSC2 mutation	TERT promoter mutation CTNNB1 mutation
Signaling pathways	mTOR, RAS-MAPK, MET IGFR1, PKA, Wnt-TGFβ AKT signaling Progenitor – IGF2, AFP, EPCAM+ Macrotrabecular – Cell cycle proliferation	IL-6 – JAK-STAT (more in steatotic type), Wnt-β-catenin signaling (more in cholestatic type)
Epigenetic features	Global DNA hypomethylation miRNA class C upregulation Progenitor type - 36 CpG signature	Cholestatic type - Extensive promoter, hypermethylation (CDKN2A, CDH1), miRNA class A, Steatotic type – miRNA class B
Immunological characteristics	Progenitor type - Immune active, Macrotrabecular type - Immune exhausted, high immune infiltrate (increased M2 macrophages intratumoral)	Steatotic type – immune active, high T-cell infiltrate of immune cells, Cholestatic type – Immune-excluded, low immune infiltrates noted
Prognosis	More aggressive, high frequency of vascular invasion, high levels of serum AFP	Less aggressive, slow-growing, low frequency of vascular invasion and low levels of AFP

Single nucleotide polymorphisms (SNPs) of patatin-like phospholipase domain-containing protein-3 (PNPLA3, rs738409), transmembrane 6 superfamily member-2 (TM6SF2, rs58542926), and hydroxysteroid 17-beta-dehydrogenase-13 (HSD17B13, rs72613567) were found to modulate the risk of chronic liver disease and HCC development, mainly in patients with alcohol-related and non-alcoholic fatty liver disease [37-38]. Even though there are no solid data to support implementing SNPs in clinical practice, as the odds ratio for using SNPs to categorize at risk for HCC is low, the role of combinations of several SNPs, called the polygenic approach, with clinical features, could prove useful for refining the prediction of risk factors, screening methods, and chemo-preventive treatment measures.

The study of mutational signatures in the development of HCC has improved our understanding of the pathogenetic mechanisms of malignant transformation. Liver tumors bear specific molecular fingerprints of exogenous (tobacco, ultraviolet alcohol, exposure to aflatoxin-B and aristolochic acid), endogenous (DNA repair mismatch, age-related changes), or sporadic processes within their genomic structure. For example, in a subgroup of HCC, mainly from Asia, the A: T to T: A transversion preferentially in a CTG trinucleotide context (called signature 22) was identified, which was found to be the hallmark of exposure to aristolochic acid [39]. Understanding the mutational signatures associated with HCC may also help in designing precision treatments. For example, in HCC harboring mutational signatures of DNA repair deficiency, based on the particular repair defect, using either DNA damaging agents, such as poly (ADP-ribose) polymerase (PARP) inhibitors (that induce synthetic lethality) or immunotherapies, may be utilized [40]. The development of HCC in a non-cirrhotic liver has been studied in two scenarios. The occurrence of HBV-related HCC in a non-fibrotic liver was demonstrated due to the action of the viral oncoprotein HBx through insertional mutagenesis. Viral insertion near a cancer gene modifies the expression or function of the gene leading to malignant transformation in the absence of cirrhosis. The main genes recurrently targeted by HBV insertions include TERT, MLL4, CCNA2, and CCNE1. Similarly, it was recently shown recurrent viral insertions of adeno-associated virus type 2 led to mono-strand defective DNA virus insertion into human DNA within the hepatocytes that resulted in malignant transformation. Thus, specific events can dictate HCC development in a non-cirrhotic liver, which forms an area of active research [41-42].

Dysbiosis, or quantitative and qualitative alterations of the gut microbiota, is well-known to be associated with chronic liver disease and cirrhosis. Recent evidence suggests the important role played by the gut microbiota in the development of HCC in cirrhosis. The disruption of the intestinal barrier, continuous exposure of the liver microenvironment to microbe-associated molecular patterns (MAMPs), and damage-associated molecular patterns, along with microbial metabolites that sustain local and systemic inflammation, as well as host antitumor immune responses, are implicated in cancer formation in the background of cirrhosis. The role of the microbiota in this regard may be dichotomous, i.e., they promote tumor formation as well as regulate antitumor responses. Gut bacterial bile acid metabolism resulting in decreased primary and increased secondary bile acids modulate liver sinusoidal cells and CXCL16-dependent natural killer T-cell recruitment as well as the senescence-associated secretory phenotype of hepatic stellate cells. Bacterial metabolites, such as trimethylamine and trimethylamine N-oxide (TMA/TMAO, generated in the presence of choline deficiency due to a change in bacterial metabolism) and (possible role of) endogenous ethyl alcohol, contribute towards hepatocarcinogenesis. Bacterial MAMPs, such as toll-like receptor (TLR)-4 agonist lipopolysaccharide and TLR-2 agonist lipoteichoic acid, act on liver stellate cells to promote the secretion of the hepato-mitogen epiregulin. The MAMPs also act on macrophages to trigger tumor-promoting inflammation as well as hepatic stellate cell activation and fibrosis, which advance HCC development [43].

The first study to profile intestinal microbiota in cirrhosis patients with and without cirrhosis demonstrated that the profile of gut microbiota associated with the former was characterized by increased fecal counts of Escherichia coli [44]. In another study in patients with hepatitis B virus-related cirrhosis and HCC, the authors found that the fecal microbial diversity increased from cirrhosis to early HCC with cirrhosis. Phylum Actinobacteria was increased, while Verrucomicrobia was reduced in early HCC compared to cirrhosis. The genera Gemmiger and Parabacteroides were enriched in early HCC compared to cirrhosis and butyrate-producing genera (beneficial) were decreased while those secreting lipopolysaccharides were increased in early HCC compared to controls. Nevertheless, in the mouse model of cirrhosis dysbiosis, a butyrate-producing inulin-rich diet promoted HCC development [45-46]. In patients with NASH-cirrhosis, those with HCC had increased levels of Bacteroides and Ruminococcaceae, whereas Akkermansia and Bifidobacterium were reduced (also inversely correlated with calprotectin concentration) when compared to those without HCC [47]. In cirrhosis patients with HBV-related HCC compared to those with HCC due to nonviral cirrhosis, the authors found that the richness of stool microbiota in the former was much higher than in healthy controls and those with nonviral related HCC. Stool from patients with nonviral HCC harbored pro-inflammatory bacteria, such as Escherichia Shigella, Enterococcus with a reduction in the levels of Faecalibacterium, and Ruminococcus, leading to the paucity of anti-inflammatory short-chain fatty acids within the luminal milieu [48].

Updates on biomarkers and their utility in HCC

Protein Biomarkers

To date, alpha-fetoprotein (AFP) is the only phase 5 biomarker (cancer control; the impact of screening on reducing the burden of disease in the population is quantified) approved for the surveillance and diagnosis of HCC in combination with ultrasonography for the former and multiphasic computed tomography or magnetic resonance imaging in the latter. The poor functionality of AFP (sensitivity for early HCC, 39% to 64%; specificity, 76% to 97%) and its ineffectiveness in accurately detecting early HCC has led to further research into identifying better biomarkers for early diagnosis during surveillance [49]. The change in AFP value over time was found to be superior to a single-point AFP value for the detection of early-stage HCC. The change in AFP was recently integrated into the validated Hepatocellular Carcinoma Early Detection Screening (including patient's current level of AFP, rate of AFP change, age, level of alanine aminotransferase, and platelet count) algorithm [50].

Prothrombin induced by vitamin K absence II (PIVKA-II or des-gamma-carboxy prothrombin, DCP), was demonstrably higher in early-stage HBV-related HCC than in chronic hepatitis B when AFP was found normal. DCP is an abnormal prothrombin formed in the presence of vitamin K deficiency, resulting from dysfunctional intracellular transport mechanisms, defects in gamma-carboxylase enzyme, and cytoskeletal changes that impair vitamin K uptake in malignant hepatocyte transformation. Higher DCP levels were also found to be associated with worse clinical behavior and shorter disease-free survival. DCP quantification suffers inconsistency in the presence of vitamin K deficiency, oral anticoagulant use, and malnourished patients with alcohol-associated cirrhosis. Recently, it was shown that DCP does not increase discriminatory power when combined with AFP and lens culinaris agglutinin-reactive glycosylated form of AFP (AFP-L3) for early HCC detection [51].

AFP-L3 was found to be insensitive in the diagnosis of HCC in patients with low-level AFP and, hence, using a better fraction method, AFP-L3% with a specificity of 85.1% was demonstrated to have better utility in patients with AFP-negative liver cancer. The greatest clinical utility of AFP-L3 or DCP has been shown to be in patients with intermediate AFP values (20-200 ng/mL) with high specificity for HCC [52].

Higher levels of osteopontin, an integrin-binding phosphoprotein that mediates cell signaling involved in regulating tumor progression, is another biomarker that was found to be associated with dedifferentiation and vascular invasion of aggressive HCC [53].

Midkine, a heparin-binding growth factor involved in cell growth, invasion, and angiogenesis during malignant transformation, was elevated in patients with very early-stage HCC [54].

Dikkopf-1, a glycoprotein that functions as a secretory antagonist of the Wnt/B-catenin signaling pathway, has a sensitivity for the detection of early-stage HCC in the range of 70%-72% with a specificity of 87%-90%. Nevertheless, preliminary data suggest that the etiology of liver disease could affect DKK1 performance as an early detection biomarker, which remains to be studied further [55].

Glypican-3, a cell surface heparan sulfate proteoglycan, was found to have a specificity of >95%, demonstrating its potential utility as a complementary biomarker to increase the sensitivity of AFP. Alpha-1 fucosidase (AFU), a lysosomal enzyme, was found to be elevated in patients with HCC, but with low specificity, as it was also shown to be overexpressed in diabetes, pancreatitis, and hypothyroidism, with variances across race and ethnicities. Nonetheless, a small study showed that the AFU activity was elevated in 85% of patients at least six months before the detection of HCC. Golgi protein-73 (GP-73), a transmembrane protein expressed in epithelial cells, identified as a potential biomarker of HCC through glycoproteomics, is also elevated in advanced fibrosis secondary to HBV or HCV infection. The squamous cell carcinoma antigen (SCCA), a serine protease inhibitor present in the squamous epithelium, is expressed by neoplastic epithelial cells and hepatocytes and has a high sensitivity for HCC but lacks specificity in differentiating HCC from cirrhosis. The fucosylated kininogen, when combined with AFP, was found to show good biomarker performance in identifying patients with early HCC. Similarly, another highly fucosylated glycoprotein called serum paraoxonase 1 (PON1) was found to be useful in the diagnosis of AFP-negative early HCC [56].

Heat shock protein 90alpha (Hsp90α) was found to be significantly elevated in liver cancer patients and positively associated with tumor staging in a large-scale multicenter clinical study [57].

Angiopoietin-like protein 2 (ANGPTL2), a secretory glycoprotein, overexpressed in HCC, was found to gradually elevate with the progression of liver injury. In patients with HBV-related liver disease, ANGPTL2 levels increased with increasing severity of fibrosis and peaked in patients with HCC associated with HBV. This was demonstrably useful for the detection of AFP-negative liver cancer [58].

The serum-detectable, cleaved, soluble extracellular portion of the transforming receptor tyrosine kinase (sAxl), a member of the tumor-associated macrophage family, was found to have sensitivity 84.6% and specificity 76.3% for the diagnosis of AFP-negative HCC with higher diagnostic performance than that of AFP for early HCC [59]. The use of high-sensitivity C-reactive protein along with AFP improved diagnostic performance with an area-under-receiver-operating-curve 0.998 and sensitivity reaching 94% [60]. High serum (enzyme-linked immunoassay (ELISA) or flow cytometric method) level of pretreatment peripheral programmed cell death ligand 1 (PPPD-L1) was associated with increased mortality as well as recurrence rate in HCC [61].

Other protein biomarkers that have been tested in phase I and phase II studies pending further validation include aldo-keto reductase family 1, member 10 (AKR1B10; cut-off value 267.9 pg/mL) with both diagnostic and prognostic value; high mobility group box 3 (HMGB3; cut-off value > 2 ng/mL) family of chromosomal proteins correlating with poor overall survival and disease-free survival; the oncofetal sal-like protein 4 (SALL4) associated with more aggressive form of HCC; serum levels of glutamine synthetase the enzyme that catalyzes glutamine synthesis, a major source of energy for tumor cells; trifucosylated N-glycan of alpha-1-acid glycoprotein (AGP) demonstrated in HCC patients but absent in healthy controls and most cirrhosis patients; autoantibodies to tumor antigens such as anti-centromere protein F and anti-heat shock protein; and more recently, serum S100P, a novel differential diagnostic marker for HCC with the potential to predict tumoral portal vein thrombus and microvascular invasion status pre-surgery [62-63].

Analysis of tissue interstitial fluid identified two overexpressed extracellular matrix proteins, SPARC and thrombospondin-2, which were valuable for the diagnosis of HCC, and patients with high thrombospondin-2 levels experienced shorter disease-free and overall survival [64]. Recently, utilizing liquid chromatography-mass spectrometry, a biomarker panel consisting of the protein metabolites, phenylalanyl-tryptophan, and glycocholate demonstrated good sensitivity in distinguishing HCC from cirrhosis and healthy controls. Furthermore, the panel was also found to have prowess in detecting AFP-negative HCC as well as solitary HCC nodules, or at most two nodules less than 3 cm in diameter. In another study, the levels of taurodeoxy cholic acid and 1,2-diacyl-3-β-d-galactosyl-sn-glycerol increased with the progression from chronic HBV-related liver disease to cirrhosis to HCC transformation, whereas the levels of 5-hydroxy-6E,8Z,11Z,14Z,17Z-eicosapentaenoic acid, and glycyrrhizic acid were found to gradually decrease with liver disease progression [65-66].

Genomic/Gene-Based Biomarkers

Dysregulated expression of circulating non-coding RNAs called microRNAs (miRNA) promotes tumorigenesis. Of these, miRNA-21 (oncogenic) and miRNA-199a (tumor suppressor) have been proposed as potential biomarkers for the early diagnosis of HCC. Similarly, the low level and downregulation of miRNA-542 and miRNA-139 were associated with poor prognosis, larger tumor size, metastatic disease, and vascular invasion. Multiple candidate miRNAs for the early detection and prognostication of HCC in genomic biomarker panels or combined with other biomarkers are under evaluation in phase I and phase II studies. Nevertheless, the lack of standardized detection protocols and data normalization across studies portend difficulty in interpreting actual evidence from miRNA-based investigations [67].

A specific type of long noncoding RNAs (lncRNA), an important regulator of carbohydrate and lipid metabolism, called the IncRNA Ftx, was found to be associated with glycolysis stimulation and HCC progression. This novel biomarker is currently studied as a prototype for early diagnosis and targeted therapy of HCC [68].

Liquid biopsy evaluation for HCC detection involves the detection of tumor-associated factors on blood samples. DNA mutations associated with HCC detected as cell-free DNA in plasma or urine have been shown to be of use in risk stratification and early cancer detection. TERT-promoter mutated plasma DNA showed 87% sensitivity for HCC detection in chronic HCV-related cirrhosis patients. Similarly, circulating cell-free methylated DNA (involved in carcinogenesis) for the early detection of HCC is undergoing validation as part of biomarker panels. The VEGF-A gene amplifications identified in circulating cell-free methylated DNA are associated with better outcomes in patients receiving sorafenib. Circulating tumor cells (CTCs) are considered potential biomarkers for the detection of HCC. Nonetheless, a recent meta-analysis concluded that a CTC assay was not recommended as an independent HCC diagnostic tool, but was strongly associated with poor clinicopathologic characteristics indicative of poor prognosis [69]. Nonetheless, liquid biopsy-related evaluations lack level 1 evidence supporting its role as a novel biomarker in HCC for clinical application.

Genetic variation, such as single nucleotide polymorphisms (SNPs) in the death receptor domain 4 gene, and the susceptibility to hepatitis C-related HCC were recently demonstrated. The A1322G SNP for the AG and GG genotype indicated a predisposition towards the development of HCC. A genome-wide association study identified an SNP variant in the gene tolloid-like 1 (TLL1) on chromosome 4, which was identified as a marker of interest in HCC risk and development. Similarly, a meta-analysis of 4528 HCC cases found that the AA allele increased the risk of developing HCC among the Asian and African populations while there was a risk of HCC with the GG allele among the Caucasian population [70-72].

Other Biomarkers and Composite Models

Apart from the role of gut microbiota in the promotion of tumorigenesis in patients with cirrhosis, the oral microbiome was found to be useful as a diagnostic biomarker for HCC. The oral microbiome, studied through 16s RNA sequencing, sampled from the tongue coat revealed that oribacterium and fusobacterium could distinguish liver cancer patients from healthy subjects. Furthermore, the microbial gene functions related to the categories of nickel/iron transport and amino acid transport were significantly different between liver cancer patients and healthy subjects [73].

The BALAD score, consisting of bilirubin, albumin, Lens-culinaris fraction of AFP, AFP, and DCP (variables in a linear relationship, six prognostic groups) and the modified BALAD-2 (variables in a continuous relationship, four prognostic groups) model categorizes the risk of liver cancer with a score > 0.24 being the highest risk for HCC development. Similar to the BALAD score, the GALAD (gender, age, AFP-L3, AFP and DCP,) model has been used to identify early HCC in patients with nonalcoholic steatohepatitis-related cirrhosis with a sensitivity of 81.4% and specificity of 89.1% [74-75]. The Doylestown algorithm, which includes log-AFP, age, gender, alkaline phosphatase, and alanine transaminase, has been demonstrated to have good prediction power for the diagnosis of early-stage HCC. In patients with low AFP (<20 ng/mL), the algorithm with the addition of fucosylated kininogen demonstrated an 89% detection rate for HCC [76].

The Hepatocellular Carcinoma Early Detection Screening (HES) algorithm, which includes age, AFP, rate of AFP change, ALT, and platelet count, was validated in a phase II study in HCV-related HCC. The algorithm was better than AFP alone in HCC detection in the six months prior to clinical diagnosis (sensitivity 53%; 10% false-positive rate) but requires validation in other nonviral etiologies of cirrhosis [77].

Machine learning methods utilizing the computational approach of relative expression ordering and maximum redundancy minimum relevance feature on microarray datasets revealed an ‘11-gene pair’ that had an outstanding ‘signature’ for the early diagnosis of HCC in a recent study [78].

**Figure 1:**
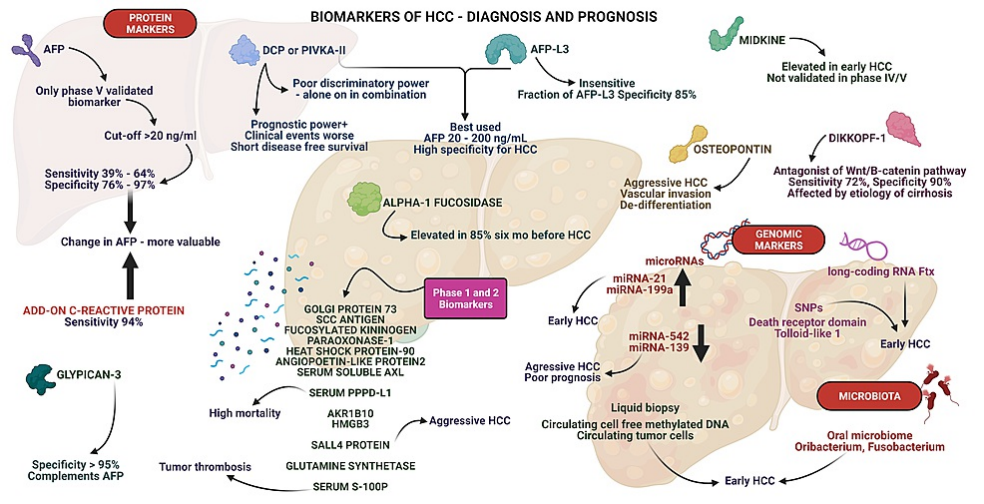
Schematic representation of biomarkers This diagram represents all the pertinent biomarkers that are currently in use and those with potential for future use in the surveillance and diagnosis of hepatocellular carcinoma. The biomarkers are divided into protein-, genome- and microbiota-based markers. AFP - alpha-fetoprotein, DCP - des-gamma-carboxy-prothrombin, PIVKA - protein induced by vitamin K absence or antagonist, SCC - squamous cell carcinoma, PPPD-L1 - pretreatment peripheral programmed cell death ligand-1, AKR1B - aldo-keto reductase 1B, HMBG - high-mobility-group-box, AFP-L3 - third electrophoretic form of lentil lectin-reactive AFP, SNP - single nucleotide polymorphism, miRNA- micro-RNA

Applying deep learning to pathological images of HCC, the authors established a ‘tumor risk score’ to evaluate patient outcomes. They found that the predictive ability of the risk score was superior to and independent of clinical staging systems and could evenly stratify patients into up to five groups with significantly different prognoses. Pathological features of sinusoidal capillarization, prominent nucleoli and karyotheca, nucleus/cytoplasm ratio, and infiltrating inflammatory cells, were the main features of the risk score [79]. The summary schematic of biomarker updates in HCC is shown in Figure 1.

Updates on imaging for HCC

Dynamic phase-contrast imaging is the standard imaging modality for the diagnosis of HCC. A recent study evaluated the diagnostic performance of contrast-enhanced computed tomography (CT) compared to MRI with extracellular contrast agents (EC-MRI) and MRI with gadoxetic acid (EOB-MRI) for HCC detection in patients with liver cirrhosis using liver explant as the reference. The authors found that EOB-MRI outperformed CT and EC-MRI for per-patient HCC detection sensitivity and was equivalent to EC-MRI for per-lesion sensitivity. MRI methods outperformed CT for the detection of HCCs sized 1-1.9 cm. Sensitive, abbreviated MRI protocols using specific contrast agents may be useful for the detection of early HCCs in cirrhosis [80]. Apart from the classic features on dynamic contrast imaging, a sharp focus on ancillary features helps in confirming the diagnosis of HCC. The presence of diffusion restriction and hypointensity on the hepatobiliary phase are suggestive of malignancy. In the presence of both features, in a hypervascular lesion without washout or an iso- or hypovascular lesion in the cirrhotic liver, the likelihood of HCC is high. Other imaging features, such as the presence of capsule-corona enhancement, fat content, mosaic architecture, and hyperintensity on T2-weighted images, favor the diagnosis of HCC. Another recent study showed that a hepatobiliary phase hypointense nodule without arterial phase hyperenhancement observed on gadoxetic acid-enhanced MRI was a strong indicator of the subsequent development of hypervascular HCC in patients with chronic liver disease [81-82].

Intravoxel incoherent motion (IVIM), a novel MRI technique, was recently used to study both diffusion and perfusion effects on liver lesions, without the need of intravenous contrast injections, useful in patients with renal impairment, contrast allergy, or to abate the long-term effects of gadolinium deposition. It can also be used to identify responders or non-responders to locoregional therapy, especially in the presence of contraindications for contrast use [83].

Diagnostic evaluation and imaging response to locoregional therapy in HCC is currently evaluated using Liver Imaging Reporting and Data Systems (LI-RADS) version 2018, which offers a comprehensive approach for a lesion-by-lesion assessment of cirrhotic nodules. In the updated version, criteria for viable and nonviable HCC and of non-evaluable tumors, as well as equivocal viability, were introduced. The viable tumor measurement obtained from the LI-RADS algorithm was utilized for patient-level response assessment via modified Response Evaluation Criteria in Solid Tumors (mRECIST; using the single largest diameter of the viable enhancing tumor during the arterial phase). The mRECIST imaging criteria were found to be a good predictor of survival and for the assessment of the antiangiogenic effect of transarterial embolotherapeutic procedures for HCC [84].

Although positron emission tomography-computed tomography (PET-CT) has very low sensitivity for the detection of smaller or well-differentiated lesions, PET-CT with 18F-fluorodeoxyglucose (18F-FDG) is useful for the useful prognostication of aggressive and poorly differentiated HCC. A recent study reported a specificity of 92% for differentiating bland from tumor thrombus using 18F-FDG PET-CT, with a mean maximum standard unit value of 4.3 for tumor thrombus. Furthermore, the presence of FDG uptake in the thrombus has been demonstrated to be a prognostic factor for overall survival and useful for the risk stratification of patients with HCC [85]. Finally, radiomics, an advanced imaging analysis in which applied computational methods for the extraction of quantitative imaging features have been utilized for identifying additional information otherwise undetectable by the human eye. For example, studies have shown that CT-based texture radiomics correctly classified bland from malignant portal vein thrombus in HCC and sensitively predicted microvascular invasion on CT imaging [86-87].

Updates on non-surgical treatments for HCC

The BCLC clinical algorithm, the most utilized staging system, provides a rationale for clinical decision-making and staging in patients with HCC. It includes prognostic, clinical, and tumor characteristics such as total bilirubin, clinically significant portal hypertension, preserved liver function, and Eastern Cooperative Oncology Group (ECOG) performance status. Other staging algorithms in use include the Cancer of the Liver Italian Program (CLIP), the Groupe d'Etude et de Traitement du Carcinome Hépatocellulaire (GRETCH) staging from France, the Chinese University Prognostic Index (CUPI), the Japan Integrated Scoring system (JIS), which integrates the Tumor-Node-Metastasis (TNM) staging with the Child-Pugh score; and recently, the Hong Kong Liver Cancer (HKLC) group system, all of which are in use in their respective regions of derivation and validation. Treatments for HCC are divided into locoregional interventions, including ablative, transarterial, and radiation therapies; systemic, including combination treatments and surgical management [88].

Locoregional therapies

Image-guided percutaneous ablative therapies are minimally invasive, potentially curative treatment options for Child-Pugh class A or B patients with BCLC stage 0 or A tumors. Available modalities in this group include chemical ablation techniques like percutaneous ethanol and acetic acid injection (PEI), and energy-based ablation techniques like radiofrequency ablation (RFA), microwave ablation (MWA), cryo/laser ablation, irreversible electroporation (IRE), and electrochemotherapy (ECT). A brief summary of ablation procedures for HCC is shown in Table 2.

**Table 2:** Characteristics of various ablative modalities used in the management of hepatocellular carcinoma

Characteristics	Radiofrequency ablation	Microwave ablation	Cryoablation	Irreversible electroporation
Energy type	Electromagnetic energy (375-500 KHz)	Electromagnetic energy (915 MHz or 2.45 GHz)	Argon-helium gas	Electric pulse (1500-3000 V/cm)
Mechanism of action	Coagulation necrosis	Coagulation necrosis	Cellular dehydration	Cell membrane disruption
Indications	Solitary or multiple tumors (fewer than 3 none more than 3 cm in size)	Solitary or multiple tumors (fewer than 3 none more than 3 cm in size)	Larger tumors close to major vascular structures, biliary trees, or other vulnerable organs	Lesions in close proximity to major vascular or biliary structures
Contraindications	Biliary obstruction, lesions in close proximity to vessels and other critical structures	Biliary obstruction, lesions in close proximity to critical structures	Coagulopathy	Patients with arrhythmias, pacemakers, and epilepsy
Advantages	Well-established, ideal for small tumors, no-touch technique	Faster heating over a larger volume, less sensitive to heat sink effect	Real-time monitoring of ablation zones, less post-procedural pain	Absence of thermal necrosis, does not affect adjoining critical structures, no-touch technique
Disadvantages	Susceptible to a heat sink, tissue impedance, thermal damage to adjoining vital structures	Less predictable and elliptical zone of ablation, thermal damage to adjoining vital structures	Usually requires multiple probes, increased bleeding risk, and risk of cryoshock	Multiple probes, a requirement of general anesthesia and deep muscular blockade, limited data on efficacy

The majority of the guidelines now recommend PEI only in cases where other ablative techniques cannot be used due to reasons like enterobiliary reflux and adhesion to the gastrointestinal tract. For tumors 2-3 cm in size, RFA is an acceptable alternative to resection. The main predictor of RFA treatment failure is tumor size, with the threshold of 3 cm diameter defining better responses, which decline dramatically with an increase in tumor size. In general, thermal ablation is aimed at inducing a 10 mm margin around the tumor edge to remove the surrounding small, undetected microsatellites. These cancer niches are frequent in tumors >3 cm and might explain the decrease in the efficacy of local ablation beyond this size cut-off. Newer generation probes like internally cooled separable cluster electrodes (Octopus®, Starmed, South Korea) and multibipolar ablation (mbp-RFA; CelonLabPower®; Olympus-Celon, Germany) can theoretically produce larger zones of ablation in a short period of time with the no-touch technique. Similarly, dual-channel RF-generators with high power, multiple switching systems, cooled-wet perfused electrodes, and multi-tuned expandable electrodes have also been introduced to overcome the limitations of conventional RF systems, but adequate clinical evidence for the same is lacking [89].

Despite the apparent technical superiority of MWA over RFA, recent meta-analyses comparing percutaneous MWA and RFA demonstrated similar efficacy of the two techniques, with a trend towards better efficacy but increased complication rates in tumors >3 cm treated with MWA [90-91].

Cryoablation is based on repetitive freeze-thaw cycles in which, initially, high-pressure argon gas leads to the cooling of the metallic probe. Subsequent infusion of helium gas then causes a warming of the probe and the thawing of the tissue. These cycles cause cell death by intracellular ice crystal formation and lead to ischemia by the formation of ice crystals within the vasculature. Based on current evidence, cryoablation is an acceptable alternative to RFA and MWA in patients with large HCCs or when HCC nodules are located close to large vessels, biliary trees, or other vital structures [92].

Irreversible electroporation (IRE) is a non-thermal ablation technique that uses short pulses of high-voltage current to increase cell membrane permeability by altering the transmembrane potential, thereby disrupting the bilayer architecture and enabling molecules to cross the membrane through nanosized pores, inducing apoptotic cell death while preserving the structural integrity of the adjoining vessels and bile ducts. The overall experience with IRE is limited and its application in clinical practice is confined to select centers in patients in whom the proximity of tumors to vital structures compromises the safety of other ablative techniques [93].

Electrochemotherapy is another novel ablative therapy for HCC that combines reversible electroporation with the concomitant administration of chemotherapeutic drugs. However, further studies are needed before electrochemotherapy enters routine clinical practice for the treatment of liver tumors [94].

TACE has been established as the most widely used therapeutic intervention for patients with intermediate-stage HCC - BCLC stage B. This includes conventional transarterial chemoembolization (cTACE), drug-eluting bead TACE (DEB-TACE), yttrium-90 radioembolization (Y-90 RE also called TARE), and bland embolization. In carefully selected patients, the median survival exceeds 40 months. A systematic review of TACE showed an objective response of 52.5% with mortality below 1%. Most of these deaths were due to liver failure, underlining the importance of patient selection for this therapy. DEB-TACE is at best non-inferior to cTACE in terms of survival benefit [95-96]. In the OPTIMIS study, the authors found that the proportion of patients with progressive disease increased with each subsequent TACE and response rates declined as the number of TACE sessions increased [97]. A recent prognostic model called the “6 and 12 score” was developed using treatment naïve, ideal candidates for TACE defined as Child-Pugh A-B7, performance status 0, no extrahepatic disease/portal vein thrombosis, and without portal hypertensive complications. This score stratified patients into three groups: those with scores ≤6, >6 but ≤12, and >12. The median overall survival in between groups was 49, 32, and 15.8 months, respectively [98]. In a large multinational cohort of HCC patients undergoing TACE, the hepatic arterial prognosis score (HAP, diameter of the largest HCC, serum AFP, albumin, and bilirubin) validation, the prognostic value of albumin-bilirubin (ALBI) score, and the impact of macrovascular invasion on survival were studied, followed by an expanded analysis. This resulted in the development of the TACE-predicted model which shed light on the additional prognostic power of tumor number, age, and etiology of underlying liver disease, enabling the prediction of post-TACE survival at the individual patient level - to identify those who would benefit from systemic therapy rather than radiological interventions [99].

Transcatheter arterial radioembolization (TARE) or selective internal radiation therapy (SIRT) is another evolving and promising transarterial treatment approach frequently used in patients with BCLC stage B tumors. Two distinct products for TARE - glass and resin microspheres embedded with yttrium-90 - are commercially available and appealing overall survival and tolerability in patients with HCC with PVT treated with glass microspheres have been reported. TARE failed to demonstrate a statistically significant improvement in overall survival compared with sorafenib, calling into question its utility in the BCLC-C stage and leading to a negative recommendation. However, subgroup analysis demonstrated that TARE might be better than sorafenib in patients with segmental PVT and low bilirubin. Currently, recommendations for TARE in the treatment of intermediate and advanced stage HCC are based on level 2 evidence [100-102].

SBRT is a highly conformal technique of external beam radiation therapy, delivering high radiation doses in a small number of fractions to a focal target using multiple, nonparallel radiation beams. The beams converge on the target lesion, minimizing radiation exposure to the normal liver parenchyma. In contrast to RFA/TACE treatment, patients with lesions located close to the liver surface, directly adjacent to large vessels, or portal vein thrombosis as well as patients presenting with extensive ascites are still candidates for SBRT. On the other hand, patients with lesions directly adjacent to structures with low radiation tolerance like small bowel or stomach are not ideal candidates because dose reduction may be necessary. Multiple studies have also compared the efficacy of SBRT with other locoregional therapies. A systematic review of studies comparing RFA and SBRT showed that both techniques achieved similar local control of disease, although RFA was superior in terms of overall survival. Several retrospective studies, cohort studies, and small RCTs have reported that SBRT as primary therapy for intermediate HCC can achieve a two-year local control of up to 80%. A retrospective study of TACE versus SBRT in patients with a tumor 3-8 cm in size showed comparable local tumor control and survival. Other investigations have shown superior local control for SBRT compared with TACE with lower toxicity, but similar overall survival [103-106].

Proton-beam therapy (PBT) has been applied to treat large HCC and those associated with vascular invasion safely. PBT was found to be safe and effective for HCC patients with inferior vena cava tumor thrombosis, especially those associated with a single lesion. The first phase III randomized controlled trial to evaluate the clinical outcomes of PBT vs. RFA in patients with recurrent small HCC found that PBT was not inferior and was quite safe for use. Further studies on this modality and the population that would benefit are unmet needs [107-108]. Multiple studies have explored the role of a combination of locoregional therapies in HCC. Theoretically, TACE prior to RFA will decrease the heat-sink effect and may increase the ablation zone. In addition, TACE will take care of the satellite nodules that are more common with larger lesions. A recent meta-analysis of pooled data from 928 patients showed that the use of TACE plus RFA for intermediate-stage hepatocellular carcinoma can attain higher tumor response rates, and improve survival rates than TACE alone. However, heterogeneity in the patient selection criteria, the combination of modalities used in these studies, and the varying endpoints preclude the derivation of any robust conclusions at this point in time [109].

Systemic therapies

For a whole decade, from 2007 to 2017, multiple studies validated various therapeutic options for HCC. The findings of the Sorafenib in Patients With Advanced Hepatocellular Carcinoma (SHARP study) led to sorafenib being the only drug approved for the treatment of advanced HCC. This changed when regorafenib was given second-line status for patients who progressed on sorafenib as per the Study of Regorafenib After Sorafenib in Patients With Hepatocellular Carcinoma (RESORCE) study. Multiple phase III studies followed that led to the approval of lenvatinib, as it was found to be non-inferior to sorafenib in overall survival as first-line as per the REFLECT trial. Further, cabozantinib and ramucirumab, the latter, the first biomarker selected treatment option for HCC patient population with previous exposure to sorafenib and AFP ≥400 ng/mL were approved. Nivolumab, as per the CheckMate 040/459 Study Group and pembrolizumab according to the KEYNOTE-224/240 Study Group were the first monoclonal antibodies to receive accelerated approval as second-line agents based on phase II trials in the United States [110].

Most of these drugs are small-molecule multikinase inhibitors of the vascular endothelial growth factor receptor (VEGFR) or monoclonal antibodies targeting VEGFR or programmed cell death receptor-1 (PD-1) and programmed cell death ligand 1 (PD-L1). The definitive role of single-agent immunotherapy is yet to be proven. In this regard, targeting both angiogenesis and immune checkpoint blockade was an area of interest, which later paid off. In HCC, the malignant cell escape from immune surveillance is mainly due to hypoxia within the tumor microenvironment due to an altered blood supply. Hypoxia reduces the functioning of immune effector cells and in the tumor environment, the activities of myeloid-derived suppressor cells and dendritic cells are reduced along with activation of hypoxia-inducible factor 1 alpha, which upregulates PD-L1 expression leading to the progression or recurrence of the tumor. Antiangiogenic treatments, such as sorafenib, can also induce hypoxia that furthers immune evasion of tumor cells. A study investigating the combination of the anti-PD-L1 antibody atezolizumab with the anti-VEGF antibody bevacizumab (IMbrave 150 Study Group) tested the hypothesis that bevacizumab would increase the activation of CD8+ T cells by reversing the immunosuppressive effects of VEGF, thereby inducing immunosuppressive cells such as regulatory T cells, tumor-associated macrophages, and myeloid-derived suppressor cells towards cytotoxicity against tumor cells. This combination was shown to improve overall and progression-free survival better than sorafenib and hence is currently the first line treatment for previously untreated, unresectable HCC. This probably steers sorafenib and lenvatinib as second-line therapies and the rest as third-line therapies in the management of advanced HCC [111-113]. The results of multiple studies in phases II and III are ongoing with respect to systemic therapies in combination strategies. Other novel therapeutic targets for HCC include inhibitors of transforming growth factor (TGF)-β, c-Met, also called tyrosine-protein kinase Met or hepatocyte growth factor receptor and fibroblast growth factor receptor 4 [114].

Combination of locoregional and systemic therapies

Even in the presence of evidence for a survival benefit for TACE in patients with BCLC stage B HCC, it remains a modality for palliation. TACE results in the creation of a hypoxic environment, which induces neoangiogenesis through the stimulation of VEGF and other pathways that lead to revascularization and residual or recurrent tumor growth. To address this issue, the addition of angiogenesis inhibitors, such as sorafenib, has been utilized in multiple studies. Different protocols exist such as sequential, interrupted, and continuous treatment. The continuous protocol helps minimize the antigenicity induced by TACE and maximizes potential synergy. Nevertheless, this may also increase the risk of hepatotoxicity, and it is presumed that inhibition of VEGF may result in the attenuation of the hepatic vasculature, which would interfere with the delivery of the drug agents. These concerns are avoided by interrupted or sequential scheduling at the cost of continuous synergy. Meta-analyses of TACE plus sorafenib demonstrated that combination therapy was better than TACE alone but led to more adverse events such as fatigue, hand-foot skin reaction, and diarrhea. Even though there was improved time to progression, there was no beneficial impact of the combination strategy on overall survival. Randomized controlled trials involving DEB-TACE with sorafenib were mostly negative trials, which did not demonstrate improvement in progression-free and overall survival even though subgroup analysis revealed advanced HCC patients benefitted from progression-free survival than those with intermediate HCC. The TACTICS study, a phase II trial from Japan, demonstrated the potential impact of study design on outcomes. This study reported improved progression-free survival in patients receiving TACE and sorafenib compared with TACE alone. The uniqueness of this study was that the development of new HCC amenable to further TACE was not associated with cessation of assigned therapy, leading to a longer time on sorafenib. To summarize, the addition of sorafenib to TACE does not improve clinically relevant outcomes for patients with intermediate-stage HCC and hence this strategy is currently not recommended [115-119].

Updates on surgical treatments for HCC

In a select group of HCC patients, surgical management can provide additional benefits beyond that achieved with locoregional and systemic therapies. Liver transplantation (LT) is the best curative treatment for HCC and in comparison, the 10-year recurrence-free survival after surgical resection was 22%-25% vs 50%-70% after LT. Nevertheless, the overall survival after resection in well-selected patients was similar to that achieved with LT due to the fact that LT patients die of non-tumor-related causes post-transplant. Hepatic resection is the treatment of choice in patients with HCC without cirrhosis. Nonetheless, resection for HCC in non-cirrhotic nonalcoholic fatty liver disease was associated with morbidity as high as 20%, similar to that observed in patients with cirrhosis [120]. In patients with cirrhosis, resection (major hepatectomy or resection of >3 segments) is recommended in those with a single tumor, regardless of size (if the residual liver volume is adequate; or preliminary portal vein embolization (PVE) can be performed), preserved liver function (Child-Pugh class A with total bilirubin <1 mg/dl; model for end-stage liver disease score (MELD) <9), absence of clinically significant portal hypertension as well as good performance status (ECOG score 0). Adherence to these selection criteria promotes five-year survival of ~70% and perioperative mortality of <3%. While waiting future liver remnant growth in patients with cirrhosis, locoregional therapy could be offered first to prevent tumor progression while the benefit of PVE manifest. Associating liver partition and portal vein ligation for staged hepatectomy (ALPPS) is an alternative towards a rapidly increasing future liver remnant [121].

The simultaneous radiological occlusion of both the portal inflow and the hepatic venous outflow of the planned target of resection was demonstrated to show better growth of the future liver remnants. Furthermore, even though it takes longer than three months for the contralateral segments to grow, the radioembolization has the additional advantage of curbing tumor growth and activity, which also helps in the analysis of tumor biology during the follow-up period leading to resection [122-123].

In cirrhosis patients with subtle signs of portal hypertension, such as platelet count <100,000/mL or splenomegaly, if MELD <9, then resection can be safely performed. Similarly, in the absence of clinically detectable ascites and indocyanine green retention <15% or liver stiffness by elastometry <12 kPA postoperative decompensation with minor hepatectomy is very low while stiffness values >20 kPA portend a high risk [124-125]. The use of virtual hepatectomy has improved the assessment of the volume and blood supply of the future liver remnants through three-dimensional reconstruction of the liver and associated tumor relationship with anatomical landmarks [126]. A recent retrospective registry study found that unplanned conversion from laparoscopic to open resection for HCC, especially for major hepatectomy, was associated with diminished overall survival compared to straight open resection. Laparoscopic hepatectomy for HCC (other than in non-cirrhotic minor resections) is still a matter of further refinement and research [127]. It is now widely believed that in selected patients with HCC and limited portal invasion, resection provides better survival outcomes than systemic therapy, even though a substantial number of patients will have an early recurrence of HCC at times, multifocally. To improve outcomes in this scenario, lobar radioembolization as the initial treatment in patients with limited [Vp1-3: Vp1 confined to a single segment; Vp2, anterior or posterior right portal vein, Vp3 as an invasion of the right or left portal vein; and Vp4 invasion extending into the main portal vein or contralateral branch] portal invasion who are otherwise good resection candidates were performed and only patients with radiographic evidence of persistently viable HCC at three months underwent surgical management [128].

**Figure 2:**
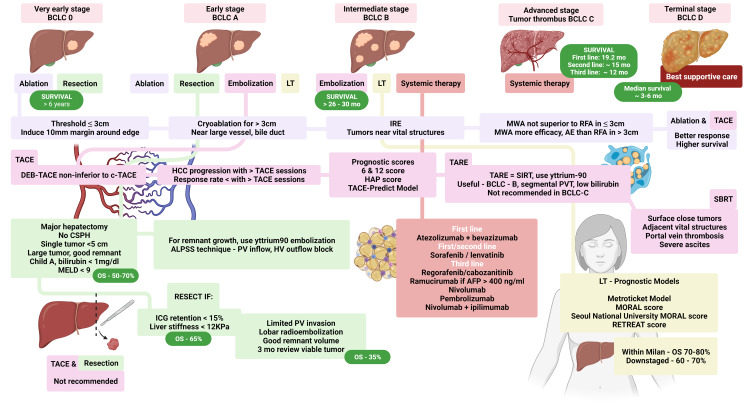
Schematic representation of updated and recommended treatment for hepatocellular carcinoma (HCC) The infographic represents the staging of HCC as per Barcelona Clinic Liver Cancer (BCLC) staging for decision-making. The overall survival (OS) associated with each treatment modality is showcased along with current updates on each of the treatment options. LT - liver transplantation, TACE - transarterial chemoembolization, TARE - transarterial radioembolization, MELD - model for end-stage liver disease, DEB-TACE - drug-eluting bead-TACE, PV - portal vein, HV - hepatic vein, ICG - indocyanine green, HAP - hepatic arterial prognostic score, MWA - microwave ablation, RFA - radiofrequency ablation, SBRT - stereotactic body radiation therapy, IRE - irreversible electroporation

Liver transplantation is recommended in HCC with advanced cirrhosis and any significant hepatic decompensation and portal hypertensive events with recurrence after LT is reported to be in the range of 11-18%. Extended selection criteria, beyond the original Milan criteria for choosing LT as the treatment of choice, have been in practice at many centers the world over, the latest being the total tumor volume (≤115 cm3)/AFP ≤400 ng/mL (TTV/AFP) model [129]. South-Asian studies have demonstrated that FDG-PET was found to be an independent predictor for HCC recurrence along with Milan criteria and AFP levels, which reveals the fact that the use of PET-based pre-transplant evaluation may help in prognostication with regard to tumor biology and postLT outcomes [130]. Many prognostic scoring models to predict outcomes in patients with HCC undergoing LT exist. These include the Metroticket Model (a sum of tumor number, size, and AFP level) for post-LT tumor recurrence and survival; the Model of Recurrence after Liver Transplant (MORAL; consisting of neutrophil: lymphocyte ratio, AFP, and tumor size, a total score between 0-13, high-risk five-year survival 18%, low risk 98.6%); Seoul National University modification of the MORAL score in hepatitis B patients, which also includes DCP and AFP levels to predict the risk of recurrence; and finally the RETREAT score (size of largest viable tumor, vascular invasion, and AFP; score 0-5 years recurrence risk <3%; score 5 risk 75%) for post-LT surveillance and need for adjuvant therapy or immunosuppression modifications (use of mTOR agents, lower risk of HCC recurrence) [131-133]. A schematic describing the current evidence for recommended therapies for HCC is shown in Figure 2.

## Conclusions

The management of HCC has seen a paradigm shift over the years due to impressive and novel evidence generation on the role of molecular biology and immune pathogenetic basis of the disease. The role of the gut microbiota in the development and progression of HCC opens new avenues for research on adjuvant therapy. Multiple novel diagnostic and surveillance biomarkers are under phased trials, and the pertinent use of biomarker panels rather than singular tests for diagnosis and prognostication has become an important area of focus. Multiple high-quality randomized trials and their systematic reviews have provided us with important information on the timing and selection of patients for potentially curative ablation or surgical resection management modalities. Such studies have also shed light on proper patient selection criteria that improve outcomes with TACE and radioembolization procedures in intermediate HCC. With the advent of immune checkpoint inhibitors and the concomitant use of antiangiogenic agents, the treatment recommendation and patient outcomes in those with advanced HCC have improved. Finally, the expansion of criteria, as well as the design of new prognostic protocols with regard to patient selection for LT, has increased the number of patients who would benefit from curative intent.
